# Barrier Properties and Characterizations of Poly(lactic Acid)/ZnO Nanocomposites

**DOI:** 10.3390/molecules25061310

**Published:** 2020-03-13

**Authors:** Zhenya Tang, Fangling Fan, Zhuangzhuang Chu, Chunli Fan, Yuyue Qin

**Affiliations:** 1 Faculty of Environmental Science and Engineering, Kunming University of Science and Technology, Kunming 650550, China; zytang@kust.edu.cn; 2 School of Energy and Environment Science, Yunnan Normal University, Kunming 650550, China; fangling.fan@outlook.com; 3 College of Materials and Energy, South China Agricultural University, Guangzhou 510642, China; 4 Institute of Agriculture and Food Engineering, Kunming University of Science and Technology, Kunming 650550, China; fanchunli1995@163.com (C.F.); rabbqy@163.com (Y.Q.)

**Keywords:** poly(lactic acid), nano-zinc oxide, barrier performances, characterizations

## Abstract

This study aimed to reinforce the barrier performance (i.e., oxygen–gas and water–vapor permeability) of poly(lactic acid) (PLA)-based films. Acetyltributylcitrate and zinc oxide nanoparticle (nano-ZnO), serving as plasticizer and nanofiller, respectively, were blended into a PLA matrix through a solvent-volatilizing method. The structural, morphological, thermal, and mechanical performances were then studied. Scanning electron microscopic images showed a significant dispersion of nano-ZnO in PLA ternary systems with low nano-ZnO content. The interaction between PLA matrix and ZnO nanoparticles was further analyzed by Fourier-transform infrared spectroscopy. Wide-angle X-ray scattering spectroscopy demonstrated high compatibility between PLA matrix and ZnO nanoparticles. Mechanical property studies revealed good tensile strength and low flexibility. Differential scanning calorimetry curves proved that an amorphous structure mostly existed in PLA ternary systems. The improvements in barrier property and tensile strength indicated that the PLA/nano-ZnO composite films could be used for food packaging application.

## 1. Introduction

Numerous biodegradable polymers had been valued in the fields of medical and food packaging over the last decade [1]. Aliphatic polyesters, such as polyglycolide acid, poly (lactic-co-glycolic acid), polyhydroxybutyrate, polycaprolactone, and poly(lactic acid) (PLA), are important biodegradable polymers [2,3,4,5]. As one of these polyesters, PLA is widely concerned with its reproducibility biodegradability, biocompatibility, moderate mechanical property, better transparency of processed materials, and low price [6,7,8]. PLA, as a linear aliphatic thermoplastic polyester, is produced by bacterial fermentation and easily biodegradable [9,10]. PLA is widely used in packaging and medical applications [11]. However, pure PLA film is brittle and rigid, and its water vapor barrier, gas barrier, and thermal stability do not meet the requirements of food packaging applications [12]. So plasticizers, polymers, and nanoparticles are frequently blended into PLA-based materials to improve the original performances of PLA [8,13,14].

The nontoxic substances used in food and drug contact materials are approved as plasticizers by relevant regulations. Plasticizers should be completely mixed with PLA and not easily evaporated during the processing of PLA composites [15]. Acetyltributylcitrate (ATBC), which is nontoxic and obtained from citric acid in nature, is usually regarded as a plasticizer for polymers used in food-packaging containers and medical and personal care applications [16]. So ATBC is considered a suitable plasticizer for PLA.

Zinc oxide nanoparticle (nano-ZnO) is a multifunctional environment-friendly inorganic nano-oxide [17]. The United States Food and Drug Administration also listed nano-ZnO as safe ‘Generally recognized as safe (GRAS)’ additive, which would not cause any destruction to the DNA of human cells [18]. The addition of nano-ZnO to polymers can improve nanocomposites characterized by a series of features, such as enhanced mechanical properties, antibacterial activity, and ultraviolet absorption [19,20,21].

In this study, PLA/ATBC-based nanocomposites with nano-ZnO in the range of 1 wt% to 15 wt% were prepared by solvent evaporation, and the barrier properties (oxygen permeability (OTR) and water vapor permeability (WVP)) of these films were evaluated. The aim of this research was to determine the changes in mechanical and barrier performances of PLA/nano-ZnO composite films, and illustrate the relationship between multiscale structure (morphological structure and crystal structure) and functional properties of food packaging films.

## 2. Results and Discussion

### 2.1. Barrier Performances

**Figure 1:**
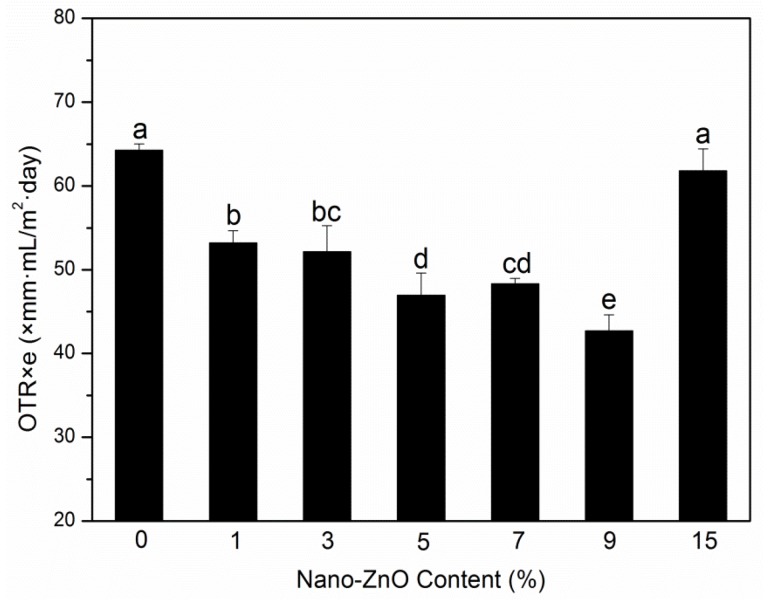
Oxygen permeability (OTR) results of poly(lactic acid) (PLA)/zinc oxide (ZnO) nanocomposite films at different contents of ZnO nanoparticles. Values with different letters in the same column were significantly differences (*p* < 0.05) among the samples.

**Figure 2:**
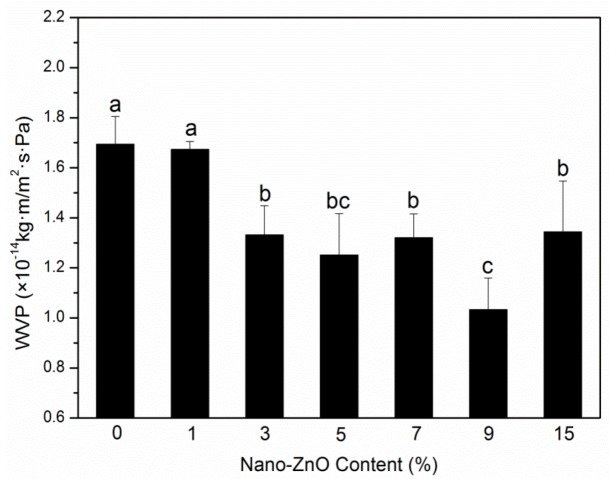
Water vapor permeability (WVP) results of PLA/ZnO nanocomposite films at different contents of ZnO nanoparticles. Values with different letters in the same column were significantly differences (*p* < 0.05) among the samples.

An important purpose of adding nanoparticle to polymer matrix is to increase their barrier performances to vapor, gases, and other substances used for food packaging. Moisture and oxygen can lead to spoilage of food, and these two substances may transfer between the internal and external atmospheres through films, following an impact on the quality of the saved products; the two barrier indexes of packaging films need to be studied [22]. The OTR values and WVP of PLA nanocomposite films are shown in Figure 1 and Figure 2, respectively.

The gas barrier properties of nanocomposite films are affected by many factors, such as the tortuous path of polymer crystal structure, the nanoparticle content, length/thickness ratio, size, type, and dispersion degree, the crystallinity of polymer matrix, and the immobilization of polymer chain [23,24,25]. The OTR values of the films are displayed in Figure 1. With an increase in nanoparticle content, the OTR values decreased. The binary system PLA-0 showed the highest value, whereas the largest reduction (33.5%) in OTR values was found in the ternary system PLA-9. The decrease in the OTR values confirmed the increase in oxygen barrier capabilities with the PLA nanocomposite films. The improved barrier properties can be related to the uniform distribution of nano-ZnO-made PLA matrix and nanoparticles that formed a strong interfacial adsorption, thereby resulting in chain immobilization and decreased diffusion of nanocomposite films [26]. The increase can also be associated with the crystallinity degree of the films. However, when increasing the nano-ZnO content to 15 wt%, the OTR value did not decrease. This result may be due to the cluster of nanoparticles in the PLA matrix, which resulted in the initialization of agglomeration and the formation of the permeability path and made the gas transfer fast.

As a rule, the tortuous path can extend the pathway of water molecule, and it is the factor that increases the water vapor barrier performance in PLA nanocomposite films [27]. The crystallinity degree, hydrophilicity, and nanoparticle content also influence water resistance. The calculated coefficients for WVP are reported in Figure 2. The WVP coefficients gradually decreased with an increase in nanoparticle concentration and showed a maximum value (1.69 × 10^−14^ kg m Pa^−1^ s^−1^ m^−2^) and a minimum value (1.03 × 10^−14^ kg m Pa^−1^ s^−1^ m^−2^) at loadings of 0 wt% and 9 wt%. This indicated an approximate tendency to the above OTR studies for binary and ternary systems. The improvement in water vapor barrier property might be because that the nanoparticles gathered in the amorphous regions of the PLA matrix, following the path for decreasing water vapor transfer. The crystallinity degree increased with increasing nano-ZnO content, which led to the improved tortuosity of the transport path [26]. The hydrophilic nano-ZnO can decrease the capability of water barrier. The overall result was that, for water vapor barrier properties, the crystallinity degree and tortuosity increased more than the hydrophilicity. For the nanoparticles with the highest loading, the coefficient (1.34 × 10^−14^ kg m Pa^−1^ s^−1^ m^−2^) was obviously higher than the PLA-9. This behavior may be due to the excessive nano-ZnO that gathered in the crystallization regions of the PLA matrix, thereby causing the compact structure to break [28]. Therefore, a good barrier performance required balanced polymer structural morphology and nanoparticle dispersion.

### 2.2. Wide-Angle X-Ray Scattering (WAXS)

**Figure 3:**
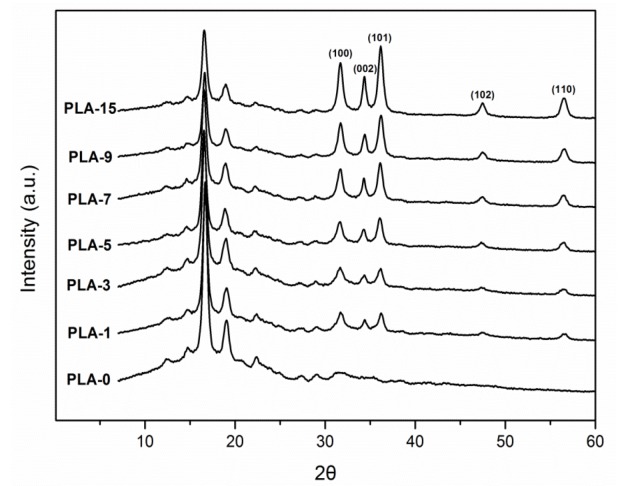
Wide-angle X-ray scattering (WAXS) patterns of PLA/ZnO nanocomposite films at different contents of ZnO nanoparticles.

The crystal structures of PLA-based nanocomposite films were characterized to analyze the influences of nanoparticle content, dispersion, and type on the crystal of the PLA matrix. Figure 3 shows the WAXS patterns of samples with different nano-ZnO contents. All samples presented a broad diffraction band at approximately 2θ = 16.8°, which demonstrated that the PLA matrix had a semi-crystalline structure. The two main peaks at 2θ = 16.7° and 19.1° were the PLA crystallization peaks that were ascribed to the crystalline plane (200) and/or (110) of the PLA typical orthorhombic crystal and crystalline plane (210), respectively [29]. In addition to the sample PLA-0, the other six nanocomposite films indicated crystalline peaks at 2θ = 31.7°, 34.4°, 36.2°, 47.5°, and 56.5°, which were ascribed to the crystal faces (100), (002), (101), (102), and (110) of the hexagonal nano-ZnO crystals, respectively [30]. Figure 3 depicts that these five significant crystalline peak intensities were apparently increasing with the addition of nanoparticles to the PLA matrix. Nano-ZnO could act as a nucleating agent to induce the formation of crystalline peaks [26]. This behavior implied that nanoparticles were embedded on the surface and inside of the PLA matrix.

### 2.3. FTIR

**Figure 4:**
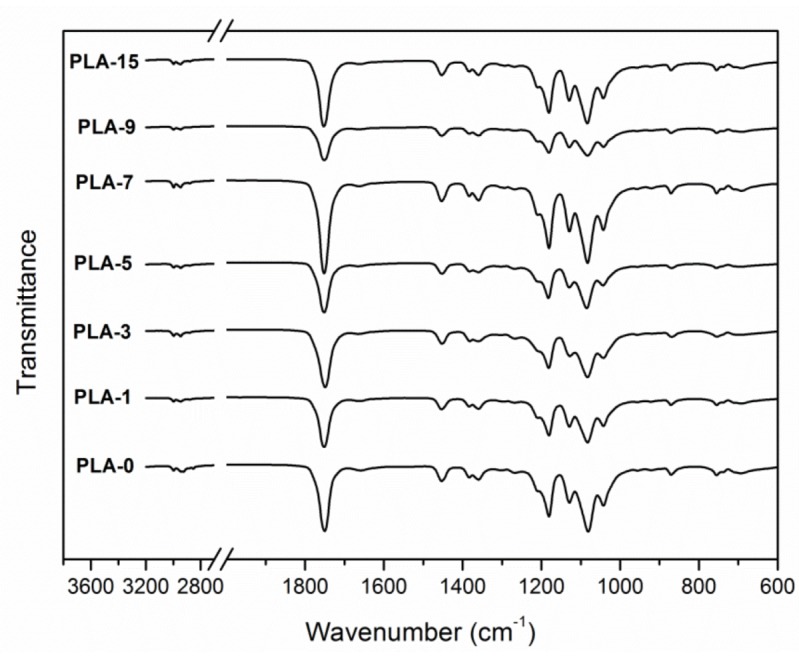
FTIR spectra of PLA/ZnO nanocomposite films at different contents of ZnO nanoparticles.

FTIR spectra are used to investigate the changes in microstructure of PLA. Figure 4 shows the FTIR spectra of PLA nanocomposite films with different nanoparticle contents. For the spectrum of PLA-0, the peak at 2996 cm^−1^ was specified to the asymmetric C-H stretching vibration of the –CH_3_ group, and that at 2945 cm^−1^ was the symmetric one. A broad and strong absorption band was attributed to the –C=O stretching vibration of esteryl at 1751 cm^−1^. The peaks at 1453 and 1383 cm^−1^ indicated asymmetric and symmetric –CH_3_ deformation vibrations, respectively. The peaks at 1181, 1129, and 1082 cm^−1^ implied asymmetric C-O-C stretching vibrations. The C-CH_3_ stretching vibration was specified to the peak at 1042 cm^−1^ [11,31,32]. The absorption bands of all samples were similar to that of the sample PLA-0, as previously reported [33]. There were slight differences in FTIR spectra of PLA-7 and PLA-9 while compared with the pure PLA film. This behavior may be because that the weak absorption peaks of nano-ZnO were masked by the strong peaks of the PLA matrix.

### 2.4. SEM

**Figure 5:**
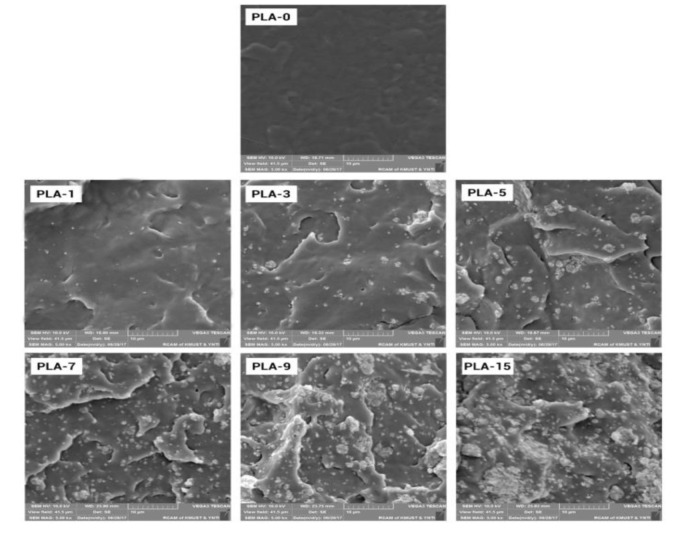
SEM images of PLA/ZnO nanocomposite films at different contents of ZnO nanoparticles (magnification: 5000).

SEM images from the low-temperature brittle fracture face of PLA-based nanocomposite films are displayed in Figure 5. From the image of the sample PLA-0 in Figure 5, a layered structure that made the polymer matrix brittle was presented. The micro-mechanisms of the nanocomposites were changed with the addition of nanoparticles to the PLA matrix. The PLA-1 film presented dispersion with homogeneous nanoparticles in the PLA matrix with little agglomerations. With an increase in the content of nanoparticles, the fractured surface became rougher, and the nanoparticles gathered to form large and small clusters. This behavior may be due to the fact that nanoparticles cannot distribute separately in the case of such high nanoparticle loading.

### 2.5. Mechanical Properties

Plasticizers and inorganic nanoparticles are often added to polymers to improve the mechanical performances of nanocomposite films. The food packaging films should be rigid and intense enough to avoid breaking during the packaging application. The mechanical performances (tensile strength (*TS*), elastic modulus (*EM*), and elongation at break (*E*)) are given in Table 1. Table 1 indicates that with an increase in the nano-ZnO content, the *TS* values of the samples gradually increased first, showing a maximum increment of 43% at the sample PLA-7 compared with that of PLA-0, and then dropped drastically. The mechanical performances of PLA-based nanocomposites were influenced by various elements, such as nanoparticle distribution, matrix crystallinity, and the interaction between polymers and nanoparticles. The increased values may be due to the random homogeneous nanoparticle dispersion and the formation of interface adhesion. When the nanoparticle content increased, a large number of clusters appeared. The nanoparticle–matrix contact surface area then decreased, hence reducing the *TS*. For the *EM* values, the trend was similar to the previous *TS* values. The nanoparticles could effectively enhance the strength and stiffness of PLA matrix. Regarding the *E* values, the trend was the opposite of the above *TS* and *EM* values. This behavior may be due to that the nanoparticles limited the ductility of PLA chain, and it may also be related to the irregular distribution and aggregation.

### 2.6. Differential Scanning Calorimetry (DSC)

**Figure 6:**
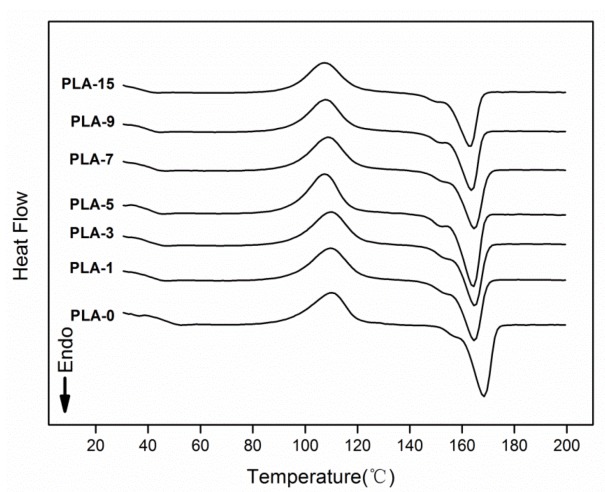
Differential Scanning Calorimetry (DSC) curves of PLA/ZnO nanocomposite films at different contents of ZnO nanoparticles.

The thermodynamic performances of the PLA-based nanocomposite films were evaluated by DSC experiments. One of the secondary calefaction curves in the three repetitions of the samples are shown in Figure 6. The thermal parameters, such as glass transition temperature (*T_g_*), cold crystallization temperature (*T_c_*), melting temperature (*T_m_*), and crystallinity degree (*X_c_*), for the films obtained from the DSC curves are summarized in Table 2. With increasing content of nanoparticles, the *T_g_* values dropped gradually. Accordingly, PLA and nano-ZnO were blended to some extent, thus making the matrix and nanoparticles change the phase transition behavior. The decrease may also be relevant to the interaction between nano-ZnO and PLA [34]. The small endothermic peaks for all samples on the curves were the result of the physical aging of the polymeric materials in glassy state [35]. As shown in Table 2, the *T_c_* and *T_m_* values gradually decreased with increasing nano-ZnO loadings. This behavior may be related to the heterogeneous nucleation. Nanoparticles acted as the heterogeneous nucleus of PLA matrix, which caused the PLA chains to crystallize at a low temperature [35]. The *X_c_* values of all samples were calculated from the DSC curves. All samples revealed low crystallinity degrees, and the sample PLA-0 had the maximum *X_c_* value of 3.4%. The amorphous structure was mostly characterized by the nanocomposite film matrix, which led to poor crystallization of films [36]. This behavior was likely caused by the interaction between the PLA matrix and the added nanoparticles mixed in the matrix [37].

### 2.7. Opacity

**Figure 7:**
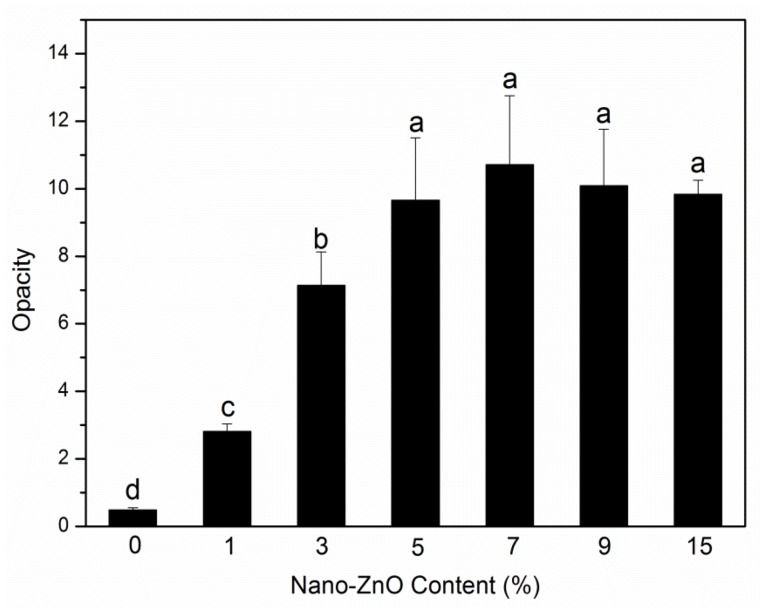
Opacity analyses of PLA/ZnO nanocomposite films at different contents of ZnO nanoparticles. Values with different letters in the same column were significantly differences (*p* < 0.05) among the samples.

**Figure 8:**
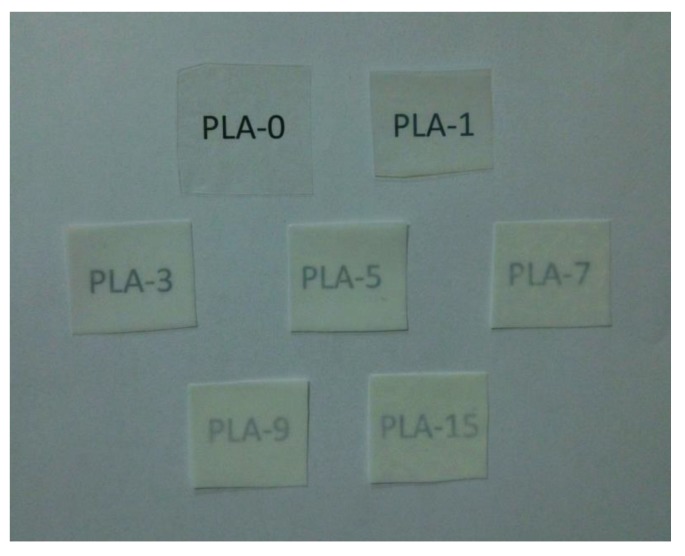
Visual appearances of PLA/ZnO nanocomposite films at different contents of ZnO nanoparticles.

The opacity of packaging materials should be considered because it is an important factor on whether consumers will purchase the packaged products. Figure 7 depicts the experimental opacity data of the samples in the absorbance of 600 nm. When the nanoparticle content was less than 7 wt%, the opacity increased rapidly with the addition of nanoparticles, which meant that the samples gradually become opaque. This behavior was mainly due to the compatibility between nanoparticles and PLA matrix [38]. The opacity was then slightly reduced, which may be due to the saturation of the nanoparticles in the PLA matrix. The above research is visually presented in Figure 8. Figure 8 shows the visual inspections of all samples. Although the nanocomposite films with more than 5 wt% of nanoparticles content had high opacity, the consumers could still see the packaged food products clearly.

## 3. Materials and Methods

### 3.1. Materials

PLA (Mw = 280 kDa, Mw/Mn = 1.98) was purchased from Natureworks LLC (Blair, NE, USA). ATBC was purchased from Shanghai Macklin Biochemical Co., Ltd. (Shanghai, China). Nano-ZnO was purchased from Qingdao Nakasen Zinc & Technology Co., Ltd. (Qingdao, Shandong, China).

### 3.2. Preparation of Nanocomposites

PLA-based nanocomposites were prepared by using the solvent evaporation method. PLA was placed in a vacuum drying chamber at 60 °C for 24 h before preparing to eliminate the effect of water. PLA, ATBC plasticizer (10 wt%), and different amounts of nano-ZnO (0, 1, 3, 5, 7, 9, and 15 wt%) were dissolved in 100 mL of dichloromethane. The solutions were stirred vigorously at room temperature until PLA and nano-ZnO were completely blended. They were then poured into polytetrafluoroethylene plates and volatilized in the same conditions. These PLA-based nanocomposites were named as PLA-0, PLA-1, PLA-3, PLA-5, PLA-7, PLA-9, and PLA-15.

### 3.3. OTR

The OTR of the films was measured with an oxygen transmittance analyzer (Oxy Sense 5250i, Oxygen Analyzer, New Castle, DE, USA). Films were cut into 6 cm × 6 cm and glued with bonding sealant in the sample chamber at 25 ± 1 °C. Nitrogen was introduced into the upper half of the sample chamber where an oxygen sensor was placed, whereas pure oxygen was fed into the below half. Each film was tested for three times, and the OTR multiplied by film thickness (OTR × *e*) was the final result.

### 3.4. WVP

The WVP (kg m Pa^−1^ s^−1^ m^−2^) of the films was calculated gravimetrically according to the ASTM E96-95 standard method [39] with proper modification. Before testing, the samples were balanced at a temperature of 25 °C and a relative humidity of 50% for 60 h. The samples were then fixed on top of weighing bottles containing desiccant. The sample bottles were moved into constant temperature and humidity. The WVP of the films was computed according to the following equation:

(1) $$\mathrm{WVP}=\frac{\Delta m\times e}{t\times A\times \Delta P}$$

where *∆m* is the weight loss of every sample test (kg), *e* is the film thickness (m), *t* is the time of every test, *A* is the test area (m^2^), and *∆P* is the vapor pressure difference (Pa) on both sides of the sample. Each film was tested for three times.

### 3.5. Wide-Angle X-ray Scattering (WAXS)

WAXS patterns of samples were obtained by using an Empyrean diffractometer with Cu K radiation, 40 kV voltage, and 40 mA currents. The samples were scanned in the range of diffraction angle 2θ from 5° to 60° by speed of 2° s^−^^1^.

### 3.6. Fourier Transform Infrared (FTIR)

FTIR experiments of the films were conducted by using an FTIR spectrometer with a wavelength range of 500 cm^−1^ to 4000 cm^−1^ with a resolution of 4 cm^−1^. Air was used as background.

### 3.7. Scanning Electron Microscopy (SEM)

SEM was conducted by using a NOVA NanoSEM 450 microscope with a scanning voltage of 10.0 kV. Before the test, the samples were immersed in liquid nitrogen for several minutes and then fractured by hand. The brittle fracture face was exposed, the samples were attached to the bracket, and gold was sprayed with 20 nm thick. The magnification was 5000 times.

### 3.8. Mechanical Performances

Tensile test was performed by using a CMT 4104 instrument according to the American Society for Testing and Materials D 882-88 standard at a speed rate of 20 mm/min with initial grip length of 50 mm at room temperature. The samples were cut into 15 mm × 100 mm. Experiments were measured in quintuplicate to obtain average values.

### 3.9. Differential Scanning Calorimetry (DSC)

DSC experiments were characterized by using DSC 214 instruments using pure nitrogen as purging gas. The samples were first heated from 20 °C to 200 °C with a heating rate of 10 °C /min, and isothermal process was conducted at this temperature for 5 min to eliminate the anterior thermal history of the samples. A subsequent cooling down to 20 °C was performed. The second heating scan from 20 to 200 °C at 10 °C/min was then used to calculate the glass-transition temperature (*T_g_*), cold crystallization temperature (*T_c_*), melting temperature (*T_m_*), and crystallinity degree (*X_c_*) for the samples. The crystallinity degree was computed according to the following equation [40]:

(2) $$X_{c}\left(\%\right)=\frac{\Delta H_{m}-\Delta H_{c}}{\Delta H_{m}^{0}\times w}\times 100$$

where *ΔH_m_* is the enthalpy of melting (J/g), *ΔH_c_* is the enthalpy of cold crystallization (J/g), *ΔH^o^_m_* is the solution heat of the full crystallization of PLA (93.7 J/g) [41], and *w* is the weight of the experiment. Repeat three times for each sample and calculate the average values.

### 3.10. Opacity

Opacity experiments were conducted with an absorbance value of 600 nm by using a UV-Vis T90 spectrophotometer [42]. The samples were cut in rectangles (7 mm × 15 mm) and fixed on a cuvette. An empty cuvette served as the control. The opacity values were computed using the following relationship:

(3) $$\mathrm{Opacity}=\frac{Abs_{600}}{e}$$

where *Ab*_*s*600_ is the absorption value of 600 nm and *e* is the sample thickness (mm). Tests were replicated three times, and average values were obtained.

## 4. Conclusions

The preparation and characterization of nanocomposite films based on PLA matrix by combining nano-ZnO and ATBC plasticizer were studied. The relationship between binary and ternary systems and barrier properties (such as water vapor permeability or oxygen permeability) of PLA nanocomposite films was analyzed. SEM images indicated uniform distribution of nanoparticles in nanocomposite films with low nano-ZnO contents, whereas some aggregations were formed in films with increasing nano-ZnO content. FTIR spectra proved the existence of interaction between PLA matrix and nano-ZnO. WAXS spectra indicated that ZnO nanoparticles showed a nucleation capability in PLA matrix, which demonstrated an apparently good compatibility between PLA matrix and ZnO nanoparticles. Mechanical property studies exhibited good tensile strength and poor flexibility. DSC curves showed that a semi-crystal structure mostly existed in PLA nanocomposite films. With regard to barrier properties, good dispersion and high crystallinity can block water and oxygen transportation. PLA/nano-ZnO composite film would be considered promising environment-friendly packaging materials.

## Figures and Tables

**Table 1:** Mechanical performances of PLA/ZnO nanocomposite films at different contents of ZnO nanoparticles.

Sample	Tensile Strength (MPa)	Elastic Modulus (MPa)	Elongation at Break (%)
PLA	20.84 ± 1.76 ^c^	1357.87 ± 185.31 ^bc^	4.06 ± 0.40 ^a^
PLA-1%	25.12 ± 2.20 ^b^	1590.72 ± 108.86 ^a^	3.70 ± 0.24 ^ab^
PLA-3%	26.76 ± 3.11 ^ab^	1601.41 ± 178.40 ^a^	3.60 ± 0.50 ^bc^
PLA-5%	27.36 ± 2.80 ^ab^	1622.36 ± 102.93 ^a^	3.55 ± 0.28 ^bcd^
PLA-7%	29.86 ± 3.07 ^a^	1680.33 ± 18.52 ^a^	3.14 ± 0.12 ^d^
PLA-9%	20.92 ± 2.00 ^c^	1523.88 ± 233.00 ^ab^	3.16 ± 0.04 ^d^
PLA-15%	16.94 ± 0.64 ^c^	1312.59 ± 68.72 ^c^	3.25 ± 0.10 ^cd^

Values with different letters in the same column were significantly differences (*p* < 0.05) among the samples.

**Table 2:** Thermal characteristics of PLA/ZnO nanocomposite films at different contents of ZnO nanoparticles.

Sample	*T_g_* (°C)	*T_c_* (°C)	*T_m_* (°C)	*X_c_* (%)
PLA-0	53.17 *±* 0.45 ^a^	111.13 *±* 0.86 ^a^	168.33 *±* 1.10 ^a^	3.60 *±* 0.20 ^a^
PLA-1	47.33 *±* 0.25 ^c^	108.83 *±* 1.33 ^bcd^	164.07 *±* 0.78 ^b^	1.48 *±* 0.06 ^e^
PLA-3	47.97 *±* 0.35 ^b^	109.70 *±* 0.70 ^ab^	163.60 *±* 1.44 ^bc^	1.90 *±* 0.03 ^d^
PLA-5	45.67 *±* 0.42 ^e^	107.77 *±* 0.57 ^cd^	162.60 *±* 1.39 ^bc^	2.11 *±* 0.07 ^c^
PLA-7	46.67 *±* 0.32 ^d^	109.87 *±* 1.16 ^ab^	162.23 *±* 2.06 ^bc^	2.25 *±* 0.05 ^c^
PLA-9	46.07 *±* 0.31 ^de^	109.43 *±* 1.46 ^abc^	161.93 *±* 1.59 ^bc^	2.46 *±* 0.06 ^b^
PLA-15	44.03 *±* 0.31 ^f^	107.10 *±* 0.61 ^d^	160.80 *±* 2.10 ^c^	1.37 *±* 0.04 ^e^

Values with different letters in the same column were significantly differences (*p* < 0.05) among the samples.
